# Supplementary material for: Efficient electrosynthesis of formamide from carbon monoxide and nitrite on a Ru-dispersed Cu nanocluster catalyst
Source: Nat Commun. 2023 May 19;14:2870. doi: 10.1038/s41467-023-38603-5 (PMC10198976; doi:10.1038/s41467-023-38603-5)
Supplement: Supplementary file 1 — Supplementary Information [file 41467_2023_38603_MOESM1_ESM.pdf]

Supplementary information

Efficient Electrosynthesis of Formamide from Carbon Monoxide and Nitrite on a Ru-dispersed Cu nanocluster catalyst

Jiao Lan[1, 4], Zengxi Wei[2, 4], Ying-Rui Lu[3, 4], DeChao Chen[1], Shuangliang Zhao[2], Ting-Shan Chan[3*], Yongwen Tan[1*]

[1]College of Materials Science and Engineering, State Key Laboratory of Advanced Design and Manufacturing for Vehicle Body, Hunan University, Changsha, Hunan 410082, China

[2]Guangxi Key Laboratory of Petrochemical Resource Processing and Process Intensification Technology and School of Chemistry and Chemical Engineering, Guangxi University, Nanning 530004, China

[3]National Synchrotron Radiation Research Center, Hsinchu 300, Taiwan

[4]These authors contributed equally: Jiao Lan, Zengxi Wei, Ying-Rui Lu.

* Corresponding author (email: chan.ts@nsrrc.org.tw, tanyw@hnu.edu.cn)

Table of Contents

1. Supplementary Figure 1 Schematic illustration of the fabrication process of TiO_2 nanowires.
2. Supplementary Figure 2 XRD patterns.
3. Supplementary Figure 3 Characterizations of Ru_1Cu SAA.
4. Supplementary Figure 4 EDS composition line profiles.
5. Supplementary Figure 5 Structure characterization of RuCu NPs.
6. Supplementary Figure 6 XPS characterization.
7. Supplementary Figure 7 Ru K-edge XANES spectra.
8. Supplementary Figure 8 Screening and optimization of structural models for Ru_1Cu SAA.
9. Supplementary Figure 9 Cu K-edge XANES spectra.
10. Supplementary Figure 10 GC spectrum.
11. Supplementary Figure 11 Standard curve of CH_3COOH quantification by ^1H NMR.
12. Supplementary Figure 12 Standard curve of $HCONH_2$ quantification by ^1H NMR.
13. Supplementary Figure 13 Standard curves of NH_3 quantification by the colouration method.
14. Supplementary Figure 14 Ru1Cu SAA CORR product detection.
15. Supplementary Figure 15 LSV curves.
16. Supplementary Figure 16 LSV curves.
17. Supplementary Figure 17 Evaluation of electrocatalytic performance.
18. Supplementary Figure 18. Comparison of electrocatalytic performance.
19. Supplementary Figure 19 Electrochemically active surface area (ECSA) tests.
20. Supplementary Figure 20 Low current density stability test.
21. Supplementary Figure 21 Hight current density stability test.
22. Supplementary Figure 22 Schematic diagram of the in-situ Raman electrolysis cell.
23. Supplementary Figure 23 NO_2^- and CO adsorption model.
24. Supplementary Figure 24 NO_2^- and CO Adsorption energies.
25. Supplementary Figure 25 Differential charge density diagram.
26. Supplementary Figure 26 Variation of NO_2^- adsorption energy on Ru_1Cu SAA with and without the help of *CO.
27. Supplementary Figure 27 Different reaction pathways of NO_2^-RR with the help of *CO.
28. Supplementary Figure 28 Different reaction pathways of NO_2^-RR without the help of *CO.
29. Supplementary Figure 29 Free energy diagram for the synthesis of formamide on the Cu NCs.
30. Supplementary Figure 30 Kinetic energy barrier diagram.
31. Supplementary Table 1 Element content analysis.
32. Supplementary Table 2 EXAFS fitting parameters.
33. Supplementary Table 3 ECSA analysis results of Cu NCs, Ru_1Cu SAA, and RuCu NPs.

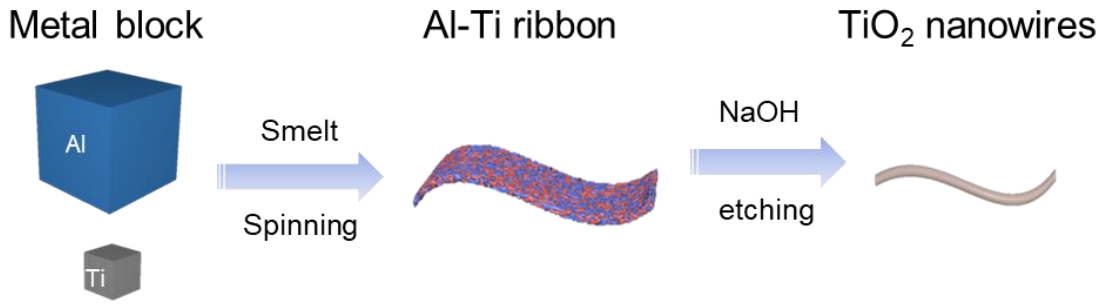

Supplementary Fig. 1| Schematic illustration of the fabrication process of TiO_2 nanowires.

Typically, the precursor $Al_{94}Ti_6$ alloy with highly-pure Al (Beijing Jiaming Platinum Nonferrous Metals Co., Ltd., 99.99%) and Ti (Beijing Jiaming Platinum Nonferrous Metals Co., Ltd.,99.99%) grain was melted with an electric arc-melting furnace in an Ar (Changsha Gaoke Gas Co., 99.999%) atmosphere. Subsequently, melt spinning technology was introduced to remelt the alloy ingot and quickly quenched on the surface of the spinning Cu roll. The rotational speed of the copper roll was 3500 rpm. To prepare the TiO_2 nanowires, these $Al_{94}Ti_6$ ribbons were completely dealloyed in NaOH (Greagent, AR) aqueous solution at room temperature. The dealloyed samples were then washed with pure water and ethanol and dried.

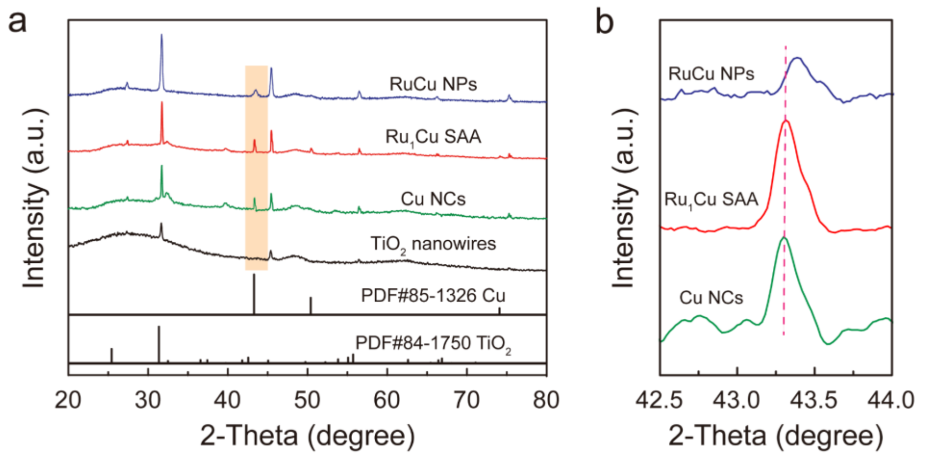

Supplementary Fig. 2| XRD patterns. XRD patterns of Precursor TiO_2 nanowires, Cu NCs, Ru_1Cu SAA and RuCu NPs.

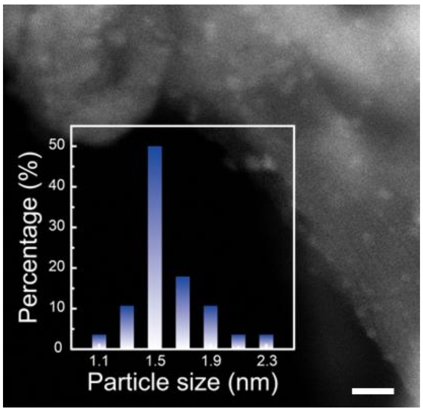

Supplementary Fig. 3| Characterizations of Ru_1Cu SAA. HAADF-STEM image of the Ru_1Cu SAA, and the corresponding nanoparticle size distribution of Ru_1Cu SAA, inset: schematic diagram of atomic structure. Scale bar: 5 nm.

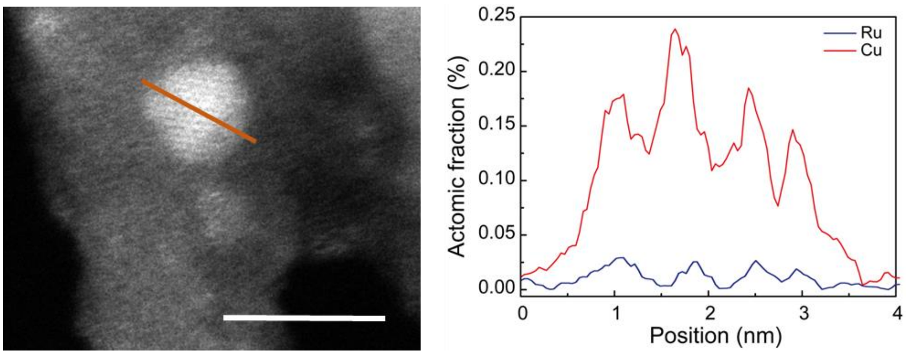

Supplementary Fig. 4| EDS composition line profiles. EDS composition line profiles of the corresponding Ru_1Cu SAA particles, Scale bar: 5 nm.

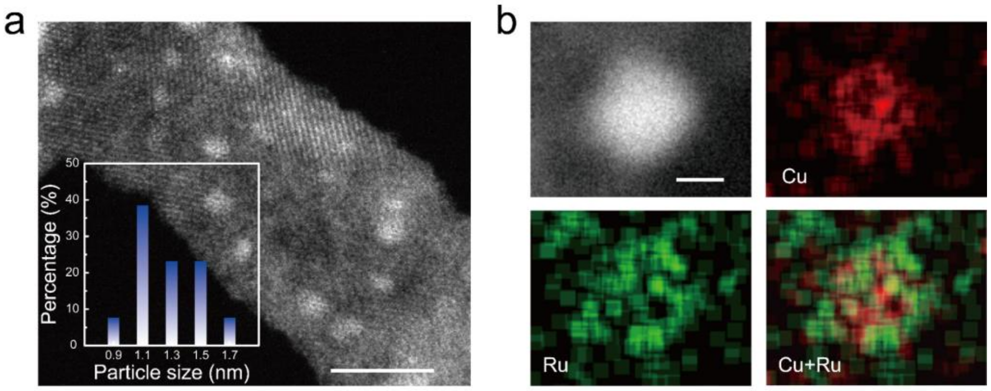

Supplementary Fig. 5| Structural characterizations of RuCu NPs. a, HAADF-STEM image of the RuCu NPs, and the corresponding nanoparticle size distribution of RuCu NPs, inset: schematic diagram of atomic structure. Scale bar: 5 nm. **b,** HAADF-STEM image and the corresponding elemental mapping. Scale bars: a 5 nm, b 1 nm.

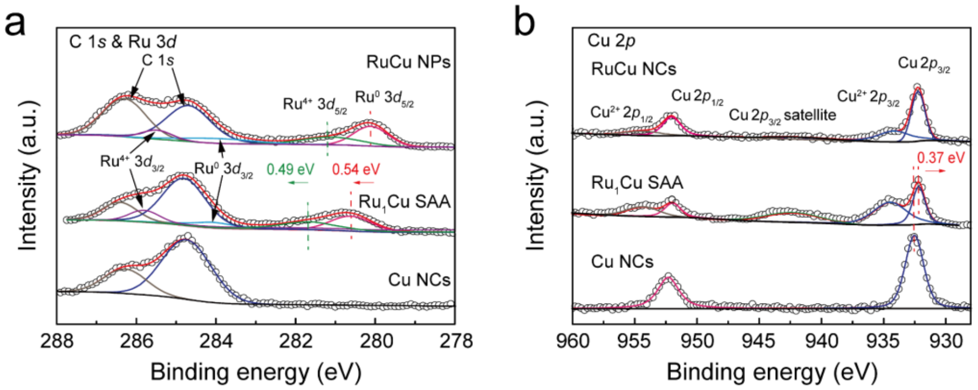

Supplementary Fig. 6| XPS characterization. C 1*s* & Ru 3*d* (**a**) and Cu 2*p* (**b**) XPS spectra of Cu NCs, Ru_1Cu SAA and RuCu NPs.

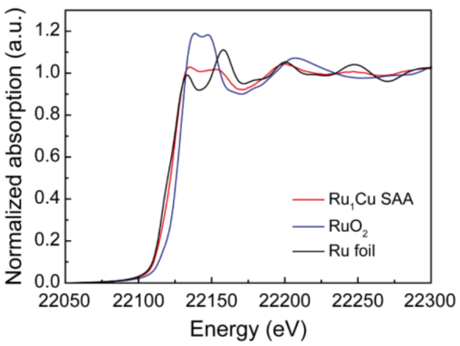

Supplementary Fig. 7| Ru K-edge XANES spectra. Ru K-edge XANES of Ru_1Cu SAA, Ru foil, and RuO_2.

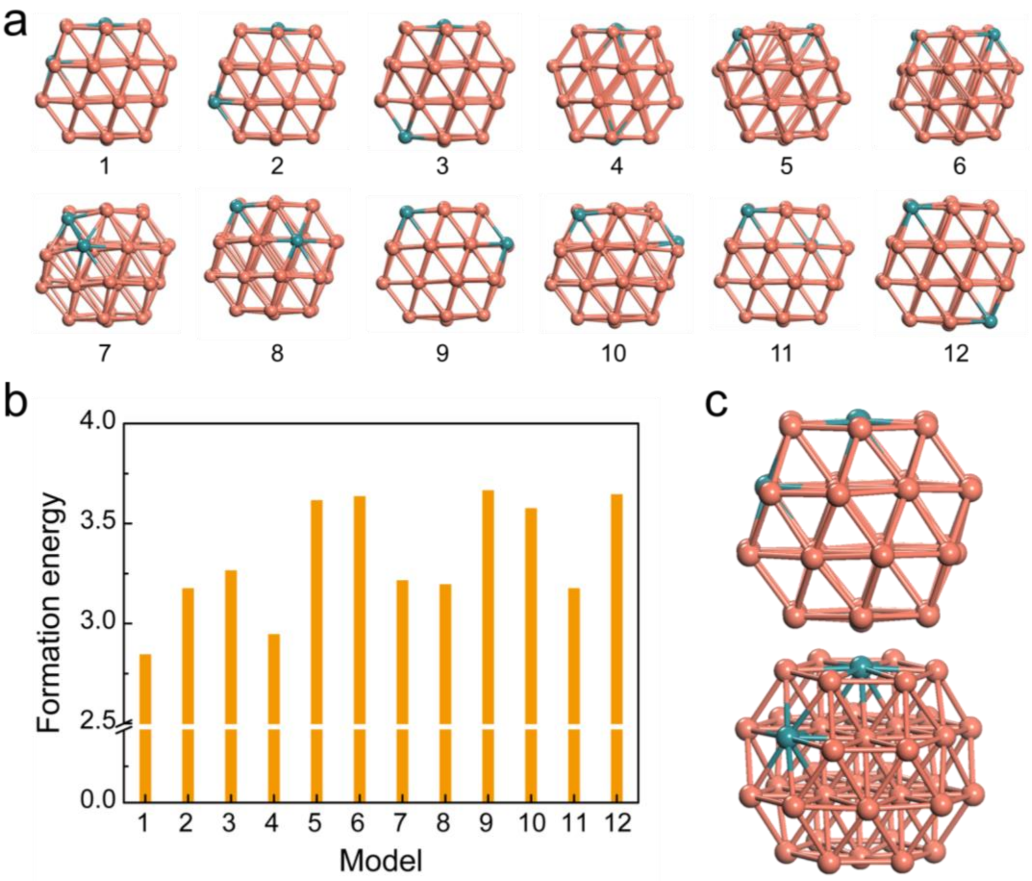

Supplementary Fig. 8| Screening and optimization of structural models for Ru_1Cu SAAa, Structural model screening of Ru_1Cu SAA. **b**, Formation energies of different Ru_1Cu SAA structures. **c**, The optimal structural model of Ru_1Cu SAA (model 1). Cu and Ru atoms shown as orange and blue, respectively

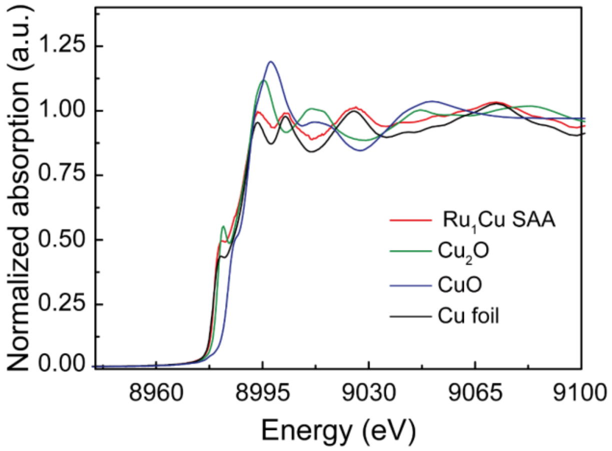

Supplementary Fig. 9| Cu K-edge XANES spectra. Cu K-edge XANES of Ru_1Cu SAA, Cu foil, Cu_2O, and CuO.

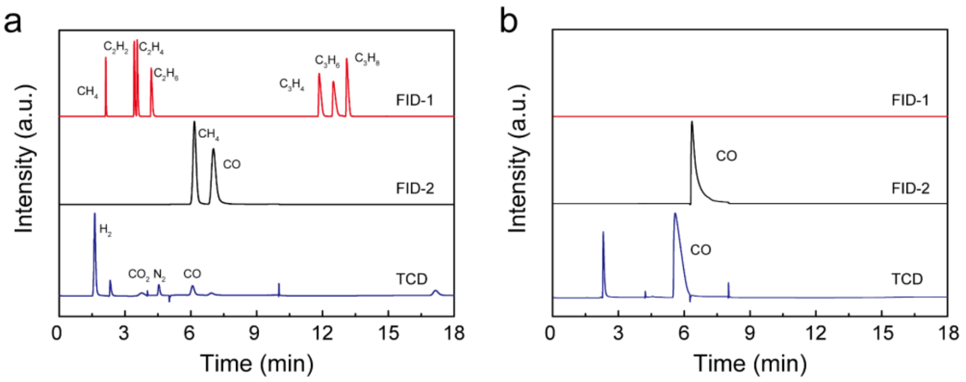

Supplementary Fig. 10| GC spectrum. a, GC spectrum of the mixed standard gas (H_2, CO, CH_4, C_2H_2, C_2H_4, et.al). **b,** GC spectrum of the high purity CO gas.

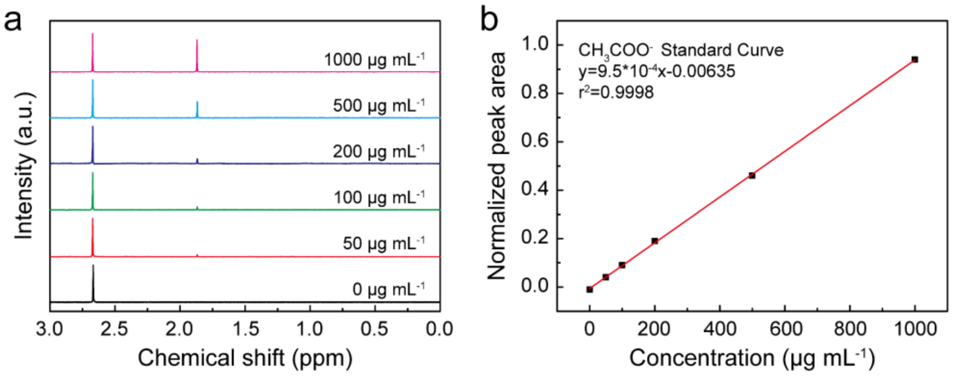

Supplementary Fig. 11| Standard curve of CH_3COOH quantification by ^1H NMR. a, ^1H NMR spectra for standard CH_3COOH with different normal concentrations. **b**, Calibration curve used for estimation of CH_3COOH.

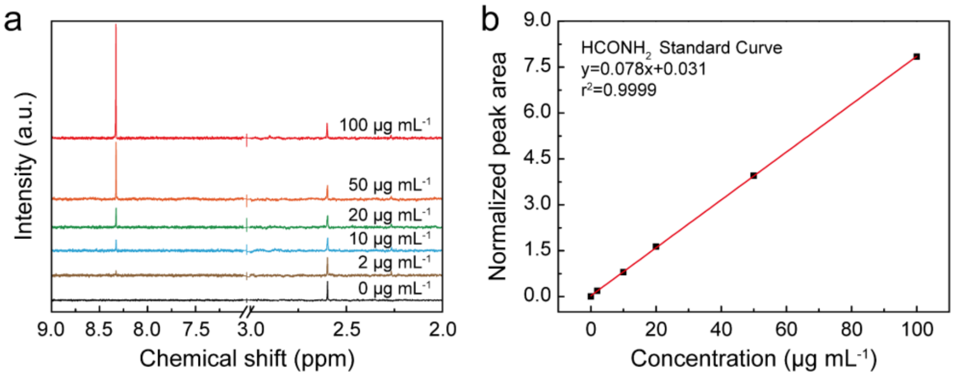

Supplementary Fig. 12| Standard curve of $HCONH_2$ quantification by ^1H NMR. a, ^1H NMR spectra for standard $HCONH_2$ with different normal concentrations. **b,** Calibration curve used for estimation of $HCONH_2$.

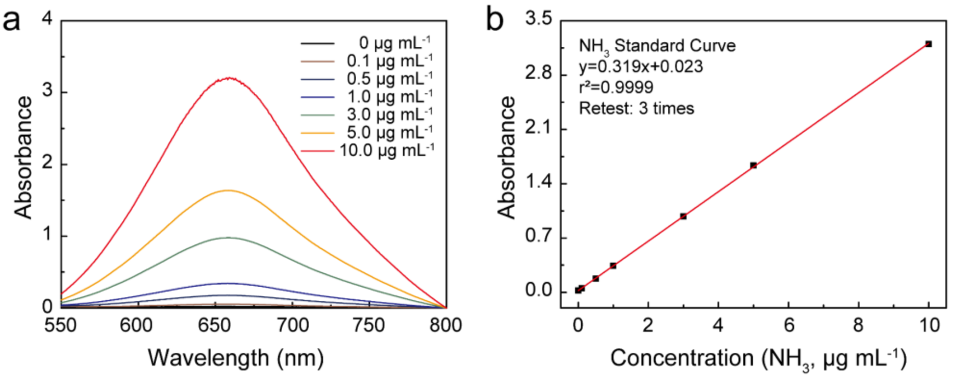

Supplementary Fig. 13| Standard curves of NH_3 quantification by the colouration method. **a,** The UV-Visible adsorption spectra of solution with different NH_3 concentrations. **b,** Calibration curve used for estimation of NH_3.

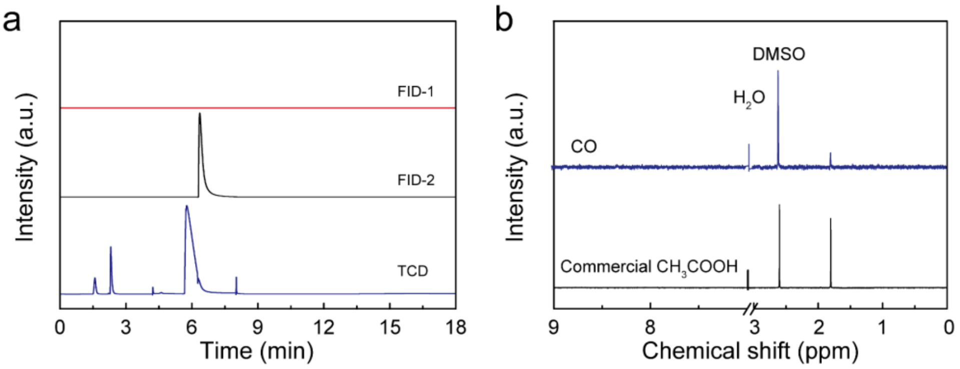

Supplementary Fig. 14| Ru_1Cu SAA CORR product detection. GC spectra of gas samples (**a**) and ^{1}H-NMR of liquid samples (**b**), which were produced on Ru_1Cu SAA catalyst after electrolysis under CO-saturated 1M KOH solution.

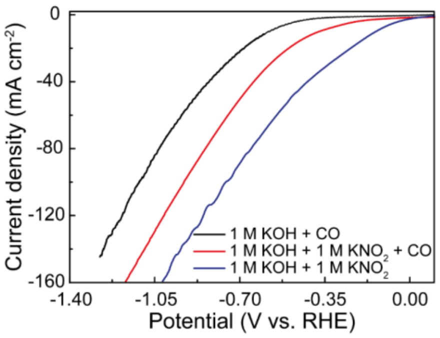

Supplementary Fig. 15| LSV curves. The LSV curves of Ru_1Cu SAA in different electrolytes.

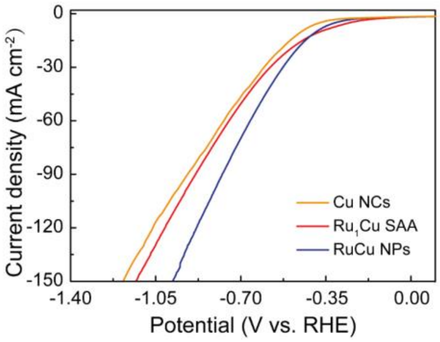

Supplementary Fig. 16| LSV curves. The LSV curves of Cu NCs, Ru_1Cu SAA and RuCu NPs in CO-saturated 1 M KOH + 1 M KNO_2 electrolyte.

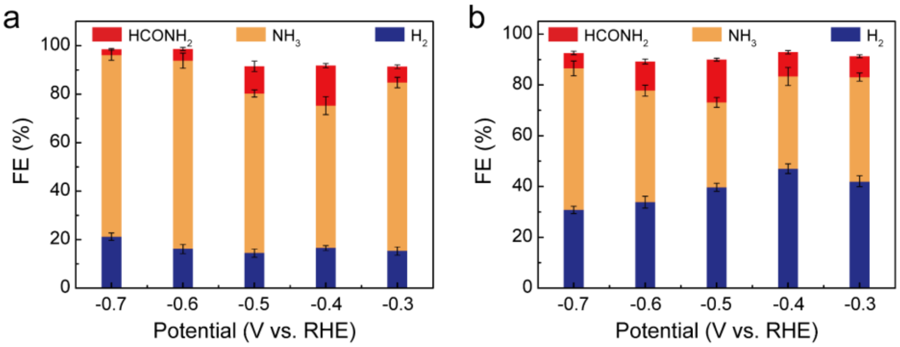

Supplementary Fig. 17| Evaluation of electrocatalytic performance. H_2, NH_3, and $HCONH_2$ Faradaic efficiency of Cu NCs (**a**) and RuCu NPs (**b**) in CO-saturated 1 M KOH + 1 M KNO_2 electrolyte at various potentials. The error bars represent the standard deviation for at least three independent measurements.

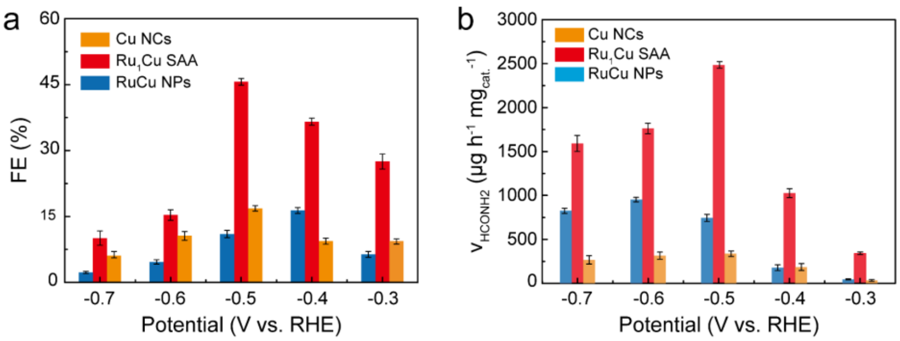

Supplementary Fig. 18| Comparison of electrocatalytic performance. a, The formamide Faradaic efficiencies of Cu NCs, Ru_1Cu SAA and RuCu NPs at various potentials. **b,** Corresponding formamide yield. The error bars represent the standard

deviation for at least three independent measurements.

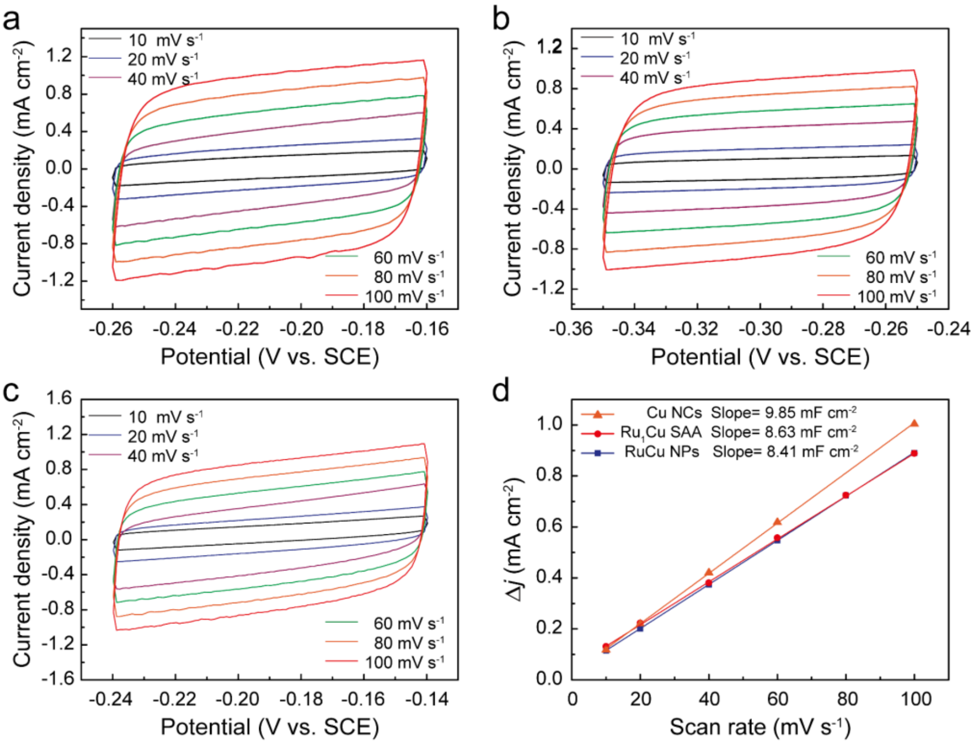

Supplementary Fig. 19| Electrochemically active surface area (ECSA) tests. Cyclic voltammograms for **a**, Cu NCs, and **b**, Ru_1Cu SAA, **c**, RuCu NPs. **d**, Plots of the current density versus the scan rate for Cu NCs, Ru_1Cu SAA and RuCu NPs. Details are demonstrated in **Supplementary Table 3**.

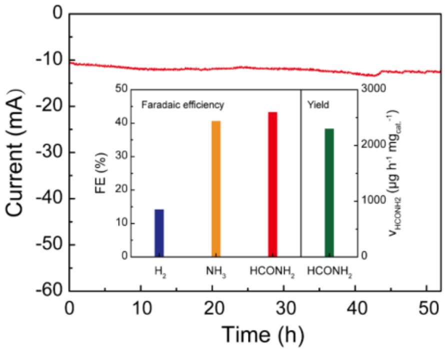

Supplementary Fig. 20| Low current density stability test. Time-dependent current curves for -0.5 V vs. RHE, inset shows the faradaic efficiencies of different products and yields of formamide after stability test.

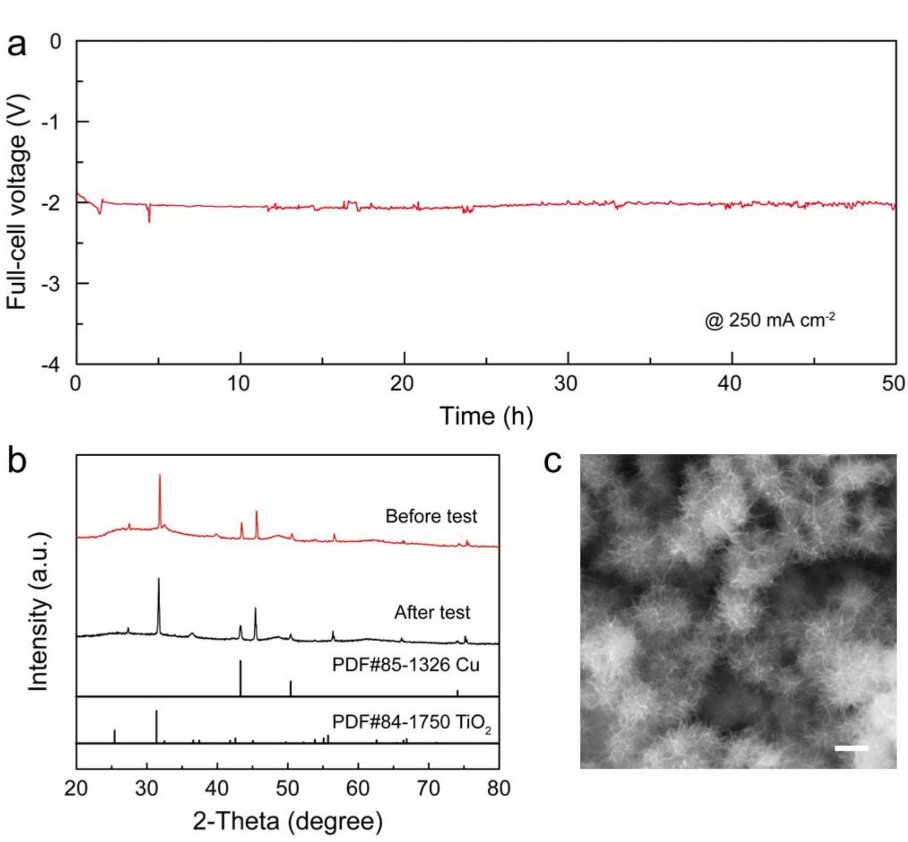

Supplementary Fig. 21| Hight current density stability test. (a) Stability test results using the MEA electrolyser at a total current density of 250 mA cm^{-2} for 50 hours. The XRD (b) and SEM (c) image after high current density stability test. Scale bar: 200 nm.

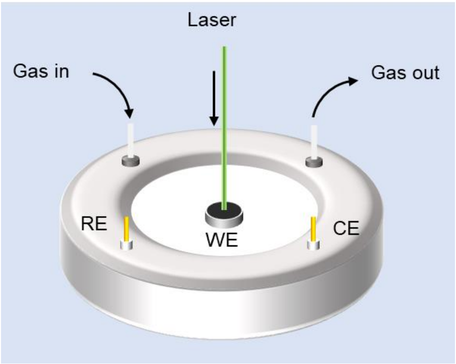

Supplementary Fig. 22| Schematic diagram of the in-situ Raman electrolysis cell.

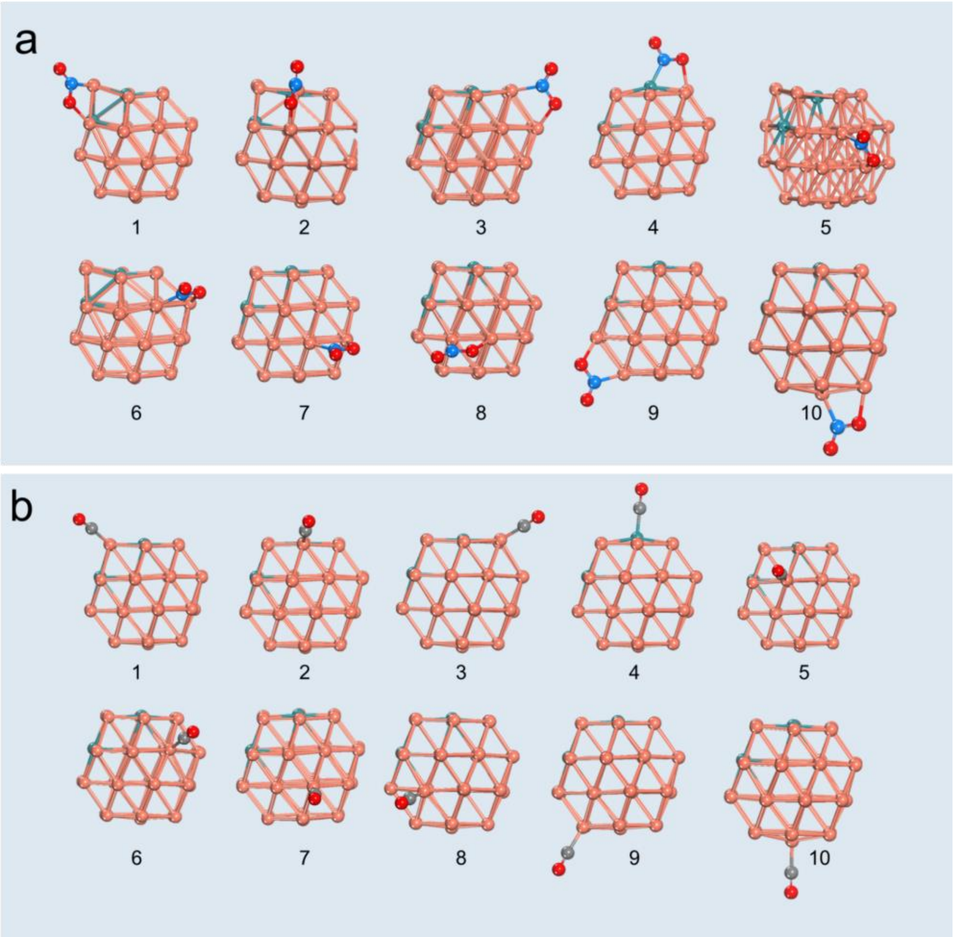

Supplementary Fig. 23| NO_2^- and CO adsorption model. Different adsorption model of NO_2^- (**a**) and CO (**b**) on the Ru_1Cu SAA model. Cu, Ru, C, O, N and H atoms shown as orange, blue, gray, red, dark blue and green, respectively.

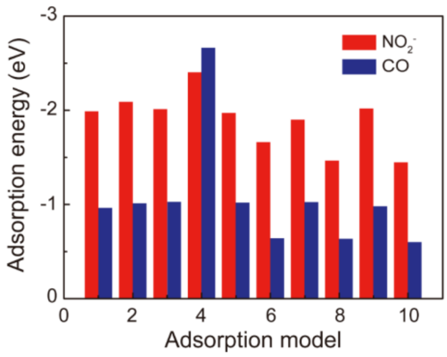

Supplementary Fig. 24| NO_2^- and CO adsorption energies. Adsorption energies of NO_2^- and CO in different adsorption modes on Ru_1Cu SAA model.

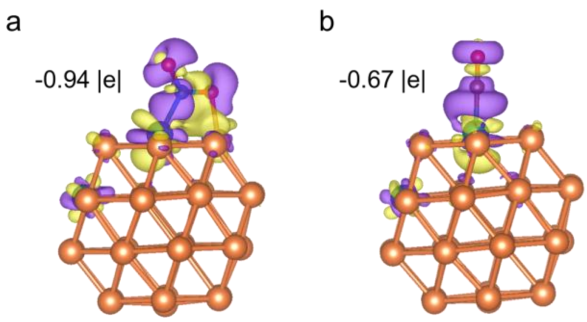

Supplementary Fig. 25. Differential charge density diagram. The electron density difference of NO_2^- (**a**) and CO (**b**) adsorbed on Ru_1Cu SAA. Cu, Ru, C, O, N and H atoms shown as orange, blue, gray, red, dark blue, and green, respectively

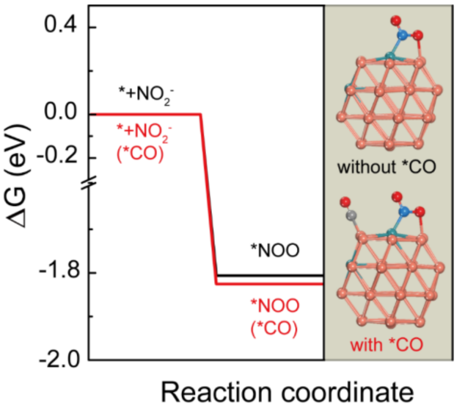

Supplementary Fig. 26| Variation of NO_2^- adsorption energy on Ru_1Cu SAA with and without the help of *CO.

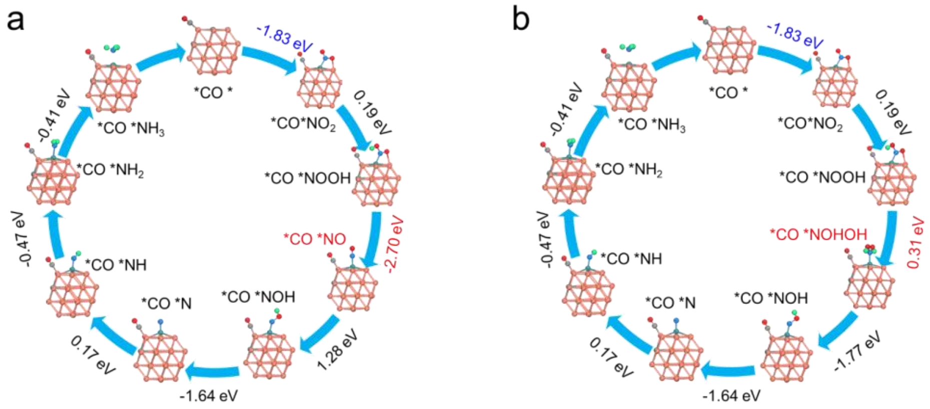

Supplementary Fig. 27| Different reaction pathways of NO_2^-RR with the help of *CO. The orange, blue, gray, red, dark blue, and green present Cu, Ru, C, O, N, and H atoms, respectively.

The adsorption process of NO_2^- on Ru_1Cu SAA surface can be regarded as the first step of the NO_2^-RR, and the value of free energy is -1.83 eV. The calculation results exhibited that the formation of the *NOOH intermediate species in first hydrogenation process requires an endothermic 0.19 eV. The third step releases 2.70 eV, if the further hydrogenation releases water and forms *NO species (path 1). Unfortunately, the subsequent hydrogenation process requires an endothermic 1.28 eV to form *NOH species, which is the rate-determining step (RDS) for NO_2^-RR. Then, the *NOH species can be attached by a proton-electron pair to form *N species and release water. Finally, the *N species undergo a three-step hydrogenation process to release ammonia. One the other hand, if the second hydrogenation process forms *NOHOH species (from *NOOH to *NOHOH), only an endothermic 0.31 eV is required, which is the RDS for NO_2^-RR (path 2). The subsequent hydrogenation process to form *NOH is exothermic (-1.77 eV). From *NOH to *N, a water is released with a free energy value of -1.68 eV. And then from the *N species to form *NH intermediate requires an endothermic 0.17 eV. The last two-step hydrogenation to ammonia process exothermic 0.47 eV and 0.41eV, respectively.

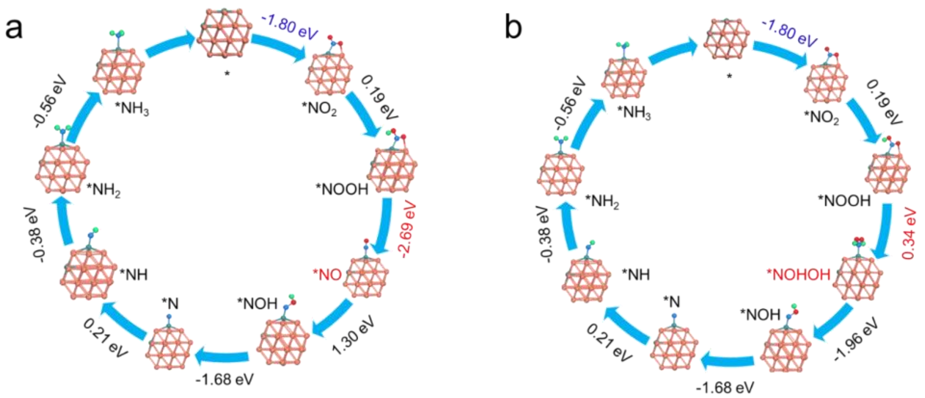

Supplementary Fig. 28| Different reaction pathways of NO_2^-RR without the help of *CO. Cu, Ru, O, N and H atoms shown as orange, blue, red, dark blue and green, respectively.

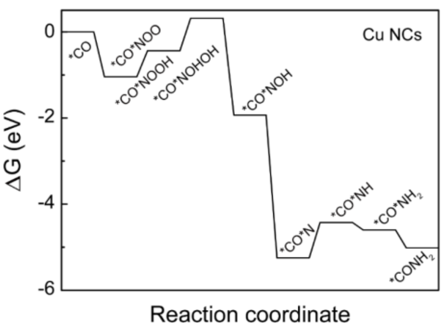

Supplementary Fig. 29| Free energy diagram for the synthesis of formamide on the Cu NCs.

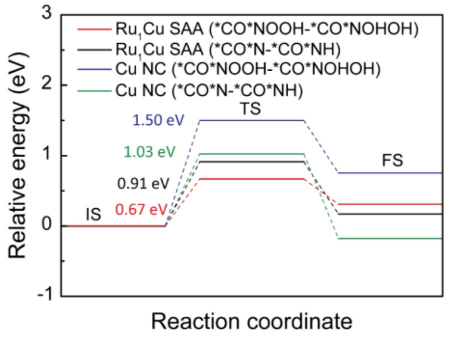

Supplementary Fig. 30| Kinetic energy barrier diagram. Kinetic energy barrier of *CO*NOHOH and *CO*NH formation on Ru_1Cu SAA and Cu NCs, respectively.

Supplementary Table 1| Element content analysis. Ru and Cu precursor concentrations (raw material concentrations) and actual concentrations (ICP-OES test results) of Ru_1Cu SAA and RuCu NPs.

sample		Cu (wt%)	Ru (wt%)	Cu:Ru (at%)
Cu NCs	precursor concentrations	4.76	-	-
	actual concentrations	5.83	-	-
Ru_1Cu SAA	precursor concentrations	4.75	0.36	95.45: 4.55
	actual concentrations	5.31	0.37	95.80: 4.20
RuCu NPs	precursor concentrations	6.82	2.33	82.32: 17.68
	actual concentrations	4.62	2.53	74.36: 25.64

Supplementary Table 2| EXAFS fitting parameters. EXAFS fitting parameters at the Ru K-edge and Cu K -edge for Ru_1Cu SAA.

	Path	*CN*	*R (Å)*	$\sigma^2 (10^{-3}$ Å$^2)$	*ΔE0 (eV)*	*R-factor*
Ru K-edge	Ru-Cu	4.8 (±0.3)	2.67 (±0.03)	6.6 (±0.3)	7.0	0.01
Cu K -edge	Cu-Cu	8.0 (±0.3)	2.53 (±0.01)	9.1 (±1.0)	4.5	0.006

Supplementary Table 3| ECSA analysis results of Cu NCs, Ru_1Cu SAA, and RuCu NPs. Slopes are calculated based on the scan rate-current relationship and their linear fitting.

	C_{dl}(mF)	ECSA (cm^{-2})
Cu NCs	9.85	246.50
Ru_1Ru SAA	8.63	215.75
RuCu NPs	8.41	210.25

The ECSA was determined by: ECSA = C_{dl}/C_s, where C_{dl} is the double layer capacitance and Cs is the specific capacitance of the sample. In this study, a general specific capacitance of Cs = 0.040 mF cm^{-2} for Cu and Ru electrode was used based on typical reported values. C_{dl} was determined by the equation: $C_{dl} = i_c/v$, where i_c is the charging current and v is the scan rate. The C_{dl} was obtained from the slopes of the linear fitting in **Supplementary Fig.19**.
